# Supplementary material for: Glutathione peroxidase 8 negatively regulates caspase‐4/11 to protect against colitis
Source: EMBO Mol Med. 2019 Nov 29;12(1):e9386. doi: 10.15252/emmm.201809386 (PMC6949489; doi:10.15252/emmm.201809386)
Supplement: Supplementary file 1 — Appendix [file EMMM-12-e9386-s001.pdf]

Glutathione peroxidase 8 negatively regulates caspase-4/11 to protect against colitis

Short Title: GPx8 negatively regulates caspase-4/11

Hus et al.

Table of contents

- **Appendix Figure S1**
- **Appendix Figure S2**
- **Appendix Figure S3**
- **Appendix Figure S4**
- **Appendix Table S1**

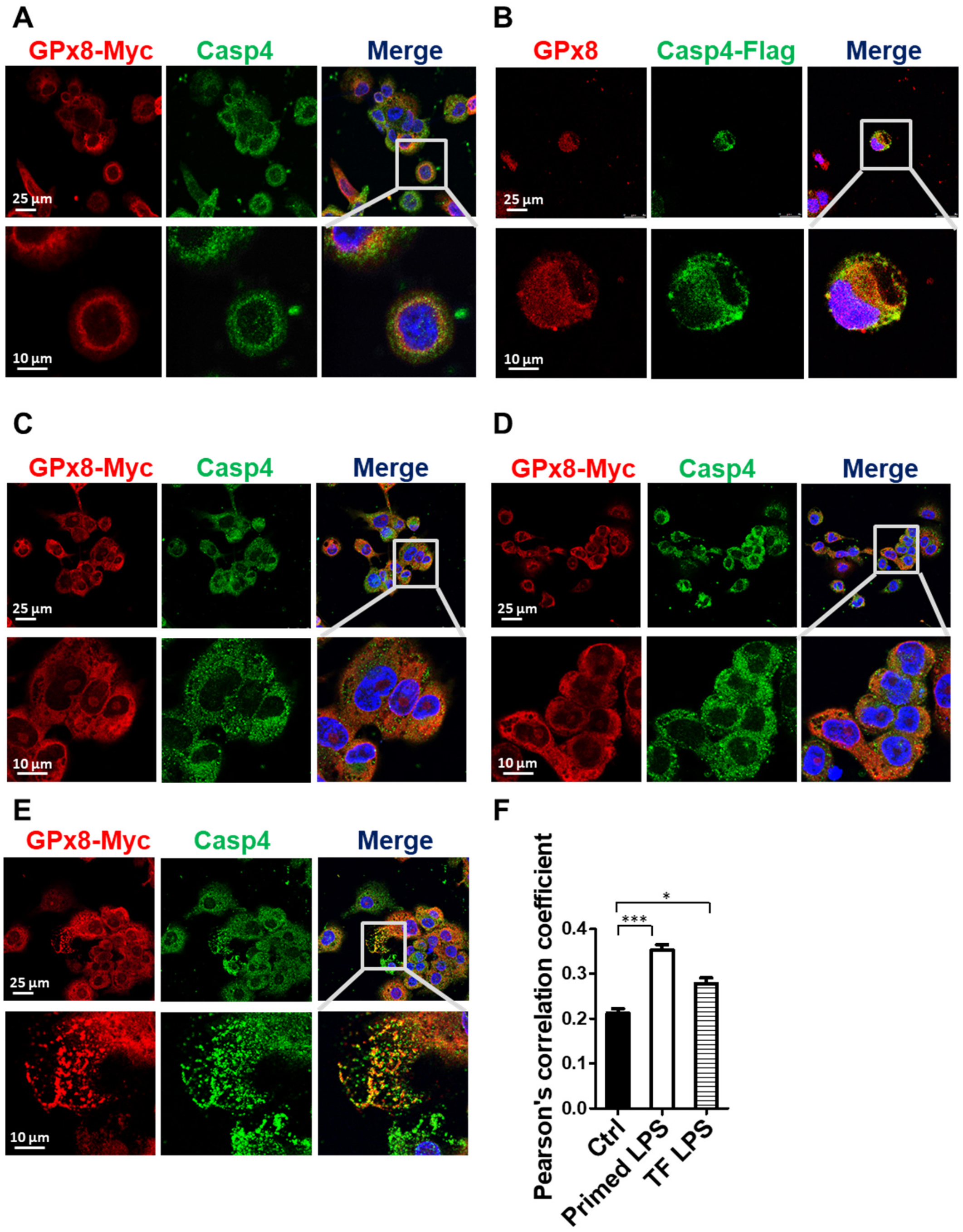

Appendix Fig S1 Co-localization of GPx8 and caspase-4 by immunofluorescence assays in THP-1 cells.(A) Figures copied from Fig. 4B show endogenous caspase-4 co-localized with Myc-tagged GPx8 (GPx8-Myc). (B) Co-localization of endogenous GPx8 with FLAG-tagged caspase-4 (Casp4-FLAG). (C-F) Co-localization of endogenous caspase-4 and Myc-tagged GPx8 (GPx8-Myc) is induced by LPS priming. Co-localization was detected in the absence (C) or presence of LPS priming for 6 h (D). (E) Co-localization was observed in THP-1 cells primed with LPS for 6 h and transfected with LPS for 4 h. (F) Co-localization was quantified by Pearson's correlation coefficient.

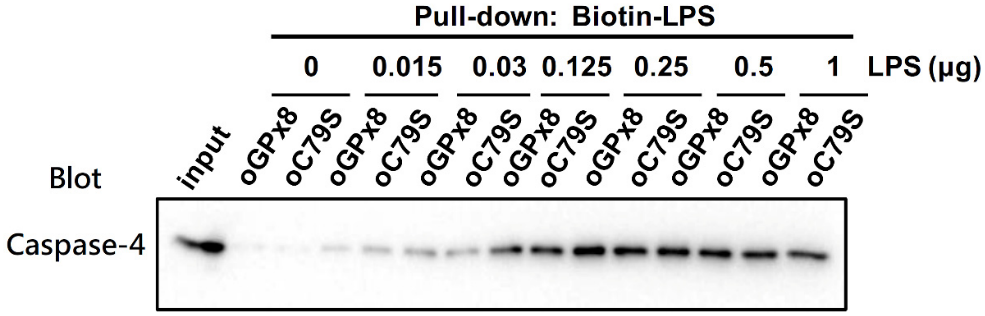

Appendix Fig S2 LPS binding activity of caspase-4 is independent of GPx8. Biotin-conjugated LPS was immobilized on streptavidin beads. Purified oxidized GPx8 (oGPx8) or GPx8C79S (oC79S) mutant proteins were co-incubated with caspase-4 for pull-down assays. LPS-bound caspase-4 was pulled down by streptavidin beads. Binding activity was demonstrated using anti-caspase-4 Ab by immunoblotting.

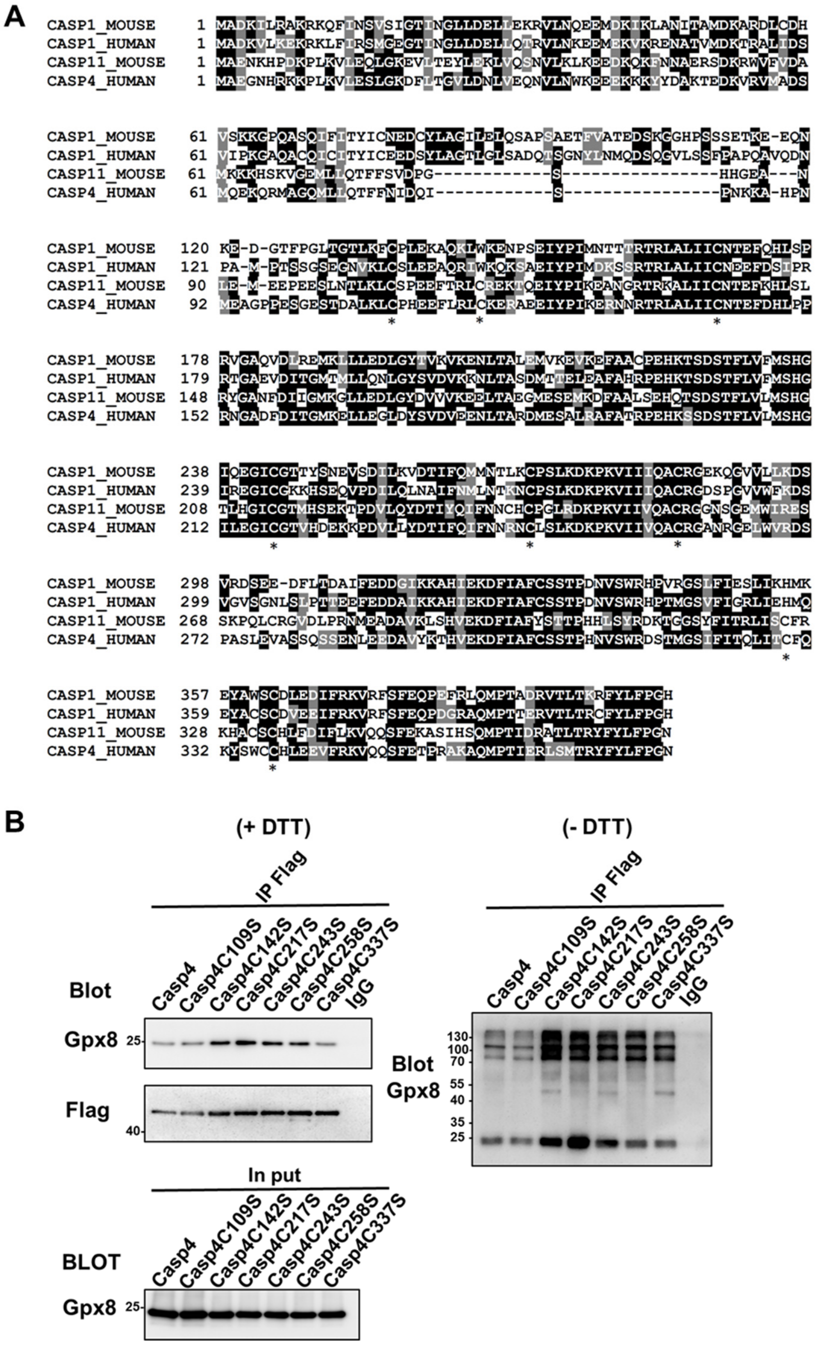

Appendix Fig S3 Identification of caspase-4 cysteine residues that interact with GPx8. (A) Conserved amino acids of caspase-1, -11 and -4 were demonstrated by sequence alignment. Asterisks indicate conserved cysteine residues present in both caspase-11 and -4. (B) Gpx8 was co-IP with FLAG-tagged WT and mutant caspase-4 proteins and subsequently analyzed by Western blots using anti-GPx8 and anti-FLAG Ab under reducing (+DTT) or non-reducing (-DTT) conditions. Disulfide-linked complexes of Gpx8 and caspase-4 are indicated.

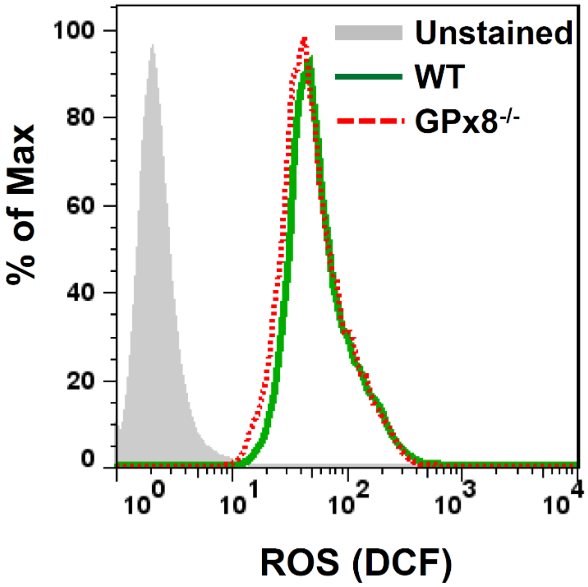

Appendix Fig S4 ROS levels in BMDMs are not altered by GPx8 deficiency under priming conditions. Quantification of endogenous ROS levels in WT and *Gpx8*$^{-/-}$ BMDMs by flow cytometry using dichlorofluorescin diacetate (DCFDA) staining. DCFDA is an ester that can be oxidized to DCF by ROS and emits green florescence upon excitation.

Appendix Table S1

Figure		p-value
1	B	P=0.0022
1	C	Day 6, P=0.02590; Day 7, P=0.00398; Day 8, P=0.00335; Day 9, P=0.02707
1	D	From Day 2 to Day 13, the p-values are as follows: 0.05342, 0.04982, 0.00023, 0.03191, 0.02105, 0.00003, 0.00018, 0.00002, 0.00264, 0.00043, 0.00059, 0.00284, 0.00062
1	E	P=0.000689
1	F	P=0.00001
1	G	P=0.011426
1	H	IL-1β, P=0.0431; IL-6, P=0.0257; IL-18, P=0.5684
1	K	Richness, P=0.0005; Chao1, P=0.0008; ACE, P=0.0008
2	C	Day 3, P=0.02322; Day 4, P=0.02591; Day 6, P=0.03398; Day 7, P=0.01037
2	D	Day 1, P=0.04566; Day 4, P=0.029939; Day 5, P=0.02145; Day 6, P=0.02235; Day 7, P=0.00002
2	E	P=0.04760
2	F	P=0.01416
2	G	P=0.03640
2	H	IL-1β, P=0.0494; IL-6, P=0.0237; IL-18, P=0.3432
3	A	TF LPS, P=6.61763E-05; MSU, P=0.44768; Nigericin, P=0.15849; ATP, P=0.08510
3	B	Left panel, TF LPS: 0.625, P= 0.01286; 1.25, P=0.0041; 2.5, P=0.00487; 5, P=0.00564<br>Right panel, TF LPS: 1.25, P=0.00008; 2.5, P=0.00003; 5, P=6.50666E-10
3	C	TF LPS: 2.5, P= 0.03550; 5, P=0.00418
3	D	Left panel: WT-EV/GPX8$^{-/-}$-EV, P=0.005776; GPX8$^{-/-}$-EV/GPX8$^{-/-}$-hGPx8, P=0.002279<br>Right panel: WT-EV/ GPX8$^{-/-}$-EV, P=4.09054E-07; GPX8$^{-/-}$-EV/GPX8$^{-/-}$-hGPx8, P=0.00092
3	G	P=0.87912
3	I	P=0.0073
3	J	P=0.0177
3	K	P=0.0292
4	C	P=6.69682E-06
4	H	EV/GPx8, P=1.94874E-08; GPx8/C79S, P=1.47297E-06; GPx8/C108S, P=0.002663559, GPx8/C2S2, P=8.72499E-06
4	I	EV/GPx8, P=0.00774; GPx8/C79S, P=7.96799E-07; GPx8/C108S, P=0.00137, GPx8/C2S2, P=0.00002
4	K	TF LPS 0.125: Mock/GPx8, P= 0.03203; GPx8/C79S, P=0.01283.<br>TF LPS 0.25: Mock/GPx8, P= 0.00281<br>TF LPS 0.5: Mock/GPx8, P= 0.04109; GPx8/C79S, P=0.00371
5	C	EV/Casp4, P=0.00009; Casp4/Casp4C258S, P=1.16852E-07; Casp4/Casp4+GPx8, P=3.83871E-07; Casp4+GPx8/Cap4C118S+GPx8, P=2.70892E-10; Casp4+GPx8/Cap4+GPx8C79S, P=3.64798E-09; Casp4+GPx8/Cap4C118S+GPx8C79S, P=4.06118E-07
5	D	EV/Casp4, P=0.00002; Casp4/Casp4C258S, P=2.86284E-05; Casp4/Casp4+GPx8, P=0.00003; Casp4+GPx8/Cap4C118S+GPx8, P=0.00001; Casp4+GPx8/Cap4+GPx8C79S, P=3.27011E-06; Casp4+GPx8/Cap4C118S+GPx8C79S, P=0.00002
6	A	TF LPS: WT/GPX8$^{-/-}$, P=0.00004<br>TF LPS+VX765:WT/GPX8$^{-/-}$, P=0.02141<br>WT-TF LPS/WT-TF LPS+VX-765, P= 2.46408E-07; GPX8$^{-/-}$-TF LPS/GPX8$^{-/-}$-TF LPS+VX-765, P= 1.98365E-07
6	B	P=0.0154
6	C	GPX8$^{-/-}$/GPX8$^{-/-}$+VX-765: Day 5, P=0.03204; Day 6, P=0.01384; Day 7, P=0.00561; Day 8, P=0.00201
6	D	From Day 1 to Day 8, the p-values of GPX8$^{-/-}$/GPX8$^{-/-}$+VX-765 are as follows: 0.00354, 0.00113, 2.83113E-09, 0.00131, 0.00001, 0.00012, 0.00392

		From Day 1 to Day 8, the p-values of WT+NAC/GPX8$^{-/-}$+NAC are as follows: 0.87057, 0.60372, 0.13896, 0.79646, 0.80825, 0.73124, 0.64876, 0.72210
6	F	IL-1β: WT/GPX8$^{-/-}$, P=0.04309; WT+VX-765/GPX8$^{-/-}$+VX-765, P=0.27940; GPX8$^{-/-}$/GPX8$^{-/-}$+VX-765, P=0.00035<br>IL-6: WT/GPX8$^{-/-}$, P=0.02572; WT+VX-765/GPX8$^{-/-}$+VX-765, P=0.22445; GPX8$^{-/-}$/GPX8$^{-/-}$+VX-765, P=0.00950
7	C	GPx8, P=0.00001; GPx7, P=0.1524; Caspase-4, P=0.0477
	D	P= 0.00432
EV1	D	P=9.36218E-08
EV2	G	TF LPS: 2.5, P=0.00031; 5, P=0.00688
EV4	A	TF LPS: WT/GPX8$^{-/-}$, P=0.00007<br>TF LPS+NAC:WT/GPX8$^{-/-}$, P=0.12809<br>WT-TF LPS/WT-TF LPS+NAC, P= 0.57092; GPX8$^{-/-}$-TF LPS/GPX8$^{-/-}$-TF LPS+NAC, P=0.00044
EV4	B	GPX8$^{-/-}$/GPX8$^{-/-}$+NAC, P=0.0101
EV4	C	GPX8$^{-/-}$/GPX8$^{-/-}$+NAC: Day 7, P=0.03864; Day8, P=0.02746
EV4	D	From Day 2 to Day 8, the p-values of GPX8$^{-/-}$/GPX8$^{-/-}$+NAC are as follows: 0.00243, 0.00002, 0.00115, 0.00001, 0.00001, 0.00044<br>From Day 2 to Day 8, the p-values of WT+NAC/GPX8$^{-/-}$+NAC are as follows: 0.58461, 0.51386, 0.04800, 0.22661, 0.90827, 0.66106, 0.18078, 0.42131
EV4	E	IL-1β: WT/GPX8$^{-/-}$, P=0.04309; WT+NAC/GPX8$^{-/-}$+NAC, P=0.66243; GPX8$^{-/-}$/GPX8$^{-/-}$+NAC, P=0.00207<br>IL-6: WT/GPX8$^{-/-}$, P=0.02572; WT+NAC/GPX8$^{-/-}$+NAC, P=0.79616; GPX8$^{-/-}$/GPX8$^{-/-}$+NAC, P=0.02029
Appendix Fig S1	F	Ctrl/Primed LPS, P=0.00098; Ctrl/TF LPS, P=0.01590
