# Supplementary material for: Glutathione peroxidase 8 negatively regulates caspase‐4/11 to protect against colitis
Source: EMBO Mol Med. 2019 Nov 29;12(1):e9386. doi: 10.15252/emmm.201809386 (PMC6949489; doi:10.15252/emmm.201809386)
Supplement: Supplementary file 2 — Expanded View Figures PDF [file EMMM-12-e9386-s002.pdf]

Expanded View Figures

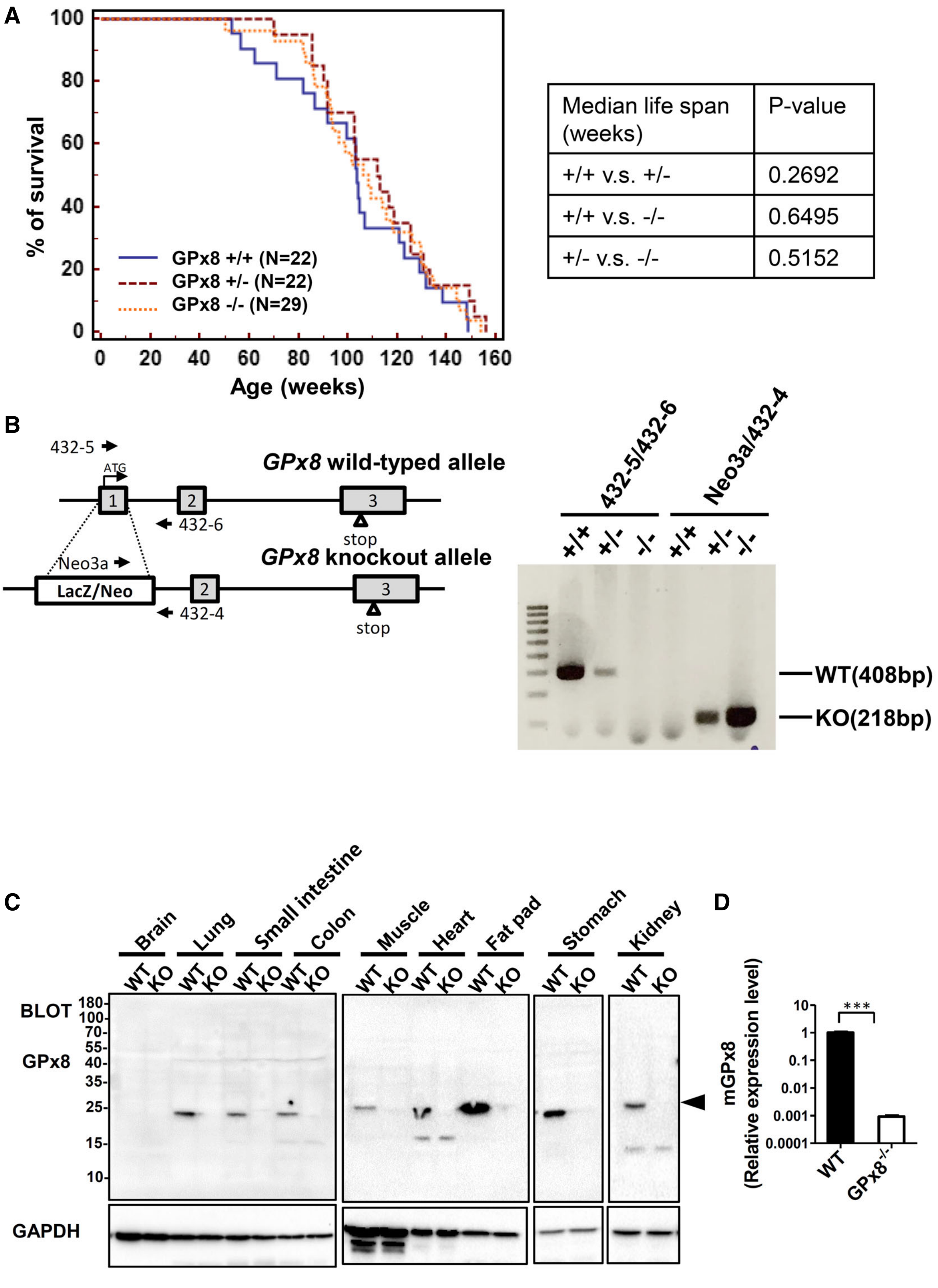

Figure EV1.

 © 2019 The Authors

Figure EV1. Characterization of *GPx8*$^{-/-}$ mice.

A *GPx8*$^{-/-}$ mice have the same life spans as WT mice.
B Genotyping of WT and *GPx8*$^{-/-}$ mice.
C Immunoblotting of GPx8 expression in various organs from WT or *GPx8*$^{-/-}$ mice. The arrowhead indicates GPx8 protein.
D Relative levels of mRNA expression for *GPx8* in WT and *GPx8*$^{-/-}$ BMDMs. Data are presented as the mean ± SD, n = 3, technical repeats. ***$P < 0.001$ (Student's two-tailed t-test).

Source data are available online for this figure.

Figure EV2. GPx8 is expressed predominantly in macrophages and does not interfere with the TLR signaling pathways.

A Tissue sections of mouse and human colons stained with anti-GPx8 Ab.
B GPx8 is expressed predominantly in macrophages and not in T, B, or dendritic cells. Cell-type specific expression of GPx8 was elucidated by immunoblotting. The black arrowhead denotes GPx8. The white arrowhead denotes non-specific bands.
C Colonic tissue from mice was stained with the pan-macrophage marker, F4/80, or dendritic marker, CD103, combined with anti-GPx8 and analyzed by confocal microscopy.
D Quantification of double-positive cells of GPx8 and CD103 or GPx8 and F4/80 was calculated from 7 fields and is presented as the mean ± SD of the percentage of double-positive cells.
E GPx8 deficiency has no effect on TLR signaling pathways. BMDMs from WT and *GPx8*$^{-/-}$ mice were primed with IFNγ (250 U/ml) and then triggered by each ligand for TLRs for 16 h. Secreted cytokines were quantified by ELISA and proportionally simplified to scoring scales as indicated.
F A flowchart of experimental procedures. BMDMs were isolated from WT or *GPx8*$^{-/-}$ mice and differentiated for 7 days. Cells were transduced with lentiviruses expressing GPx8 if needed on day 3. On day 7, BMDMs were primed with LPS or poly(I:C) for 5–6 h and then transfected with LPS for 8 h or with other activators for the indicated times.
G Enhanced non-canonical inflammasome activation in GPx8-depleted human macrophages. Human macrophages were transfected with scrambled siRNA or siGPx8 for 3 days then primed and transfected with LPS. Data are representative of at least three independent experiments and presented as the mean ± SD, n = 5, technical repeats. **$P < 0.01$; ***$P < 0.001$ (Student's two-tailed t-test).

Source data are available online for this figure.

© 2019 The Authors

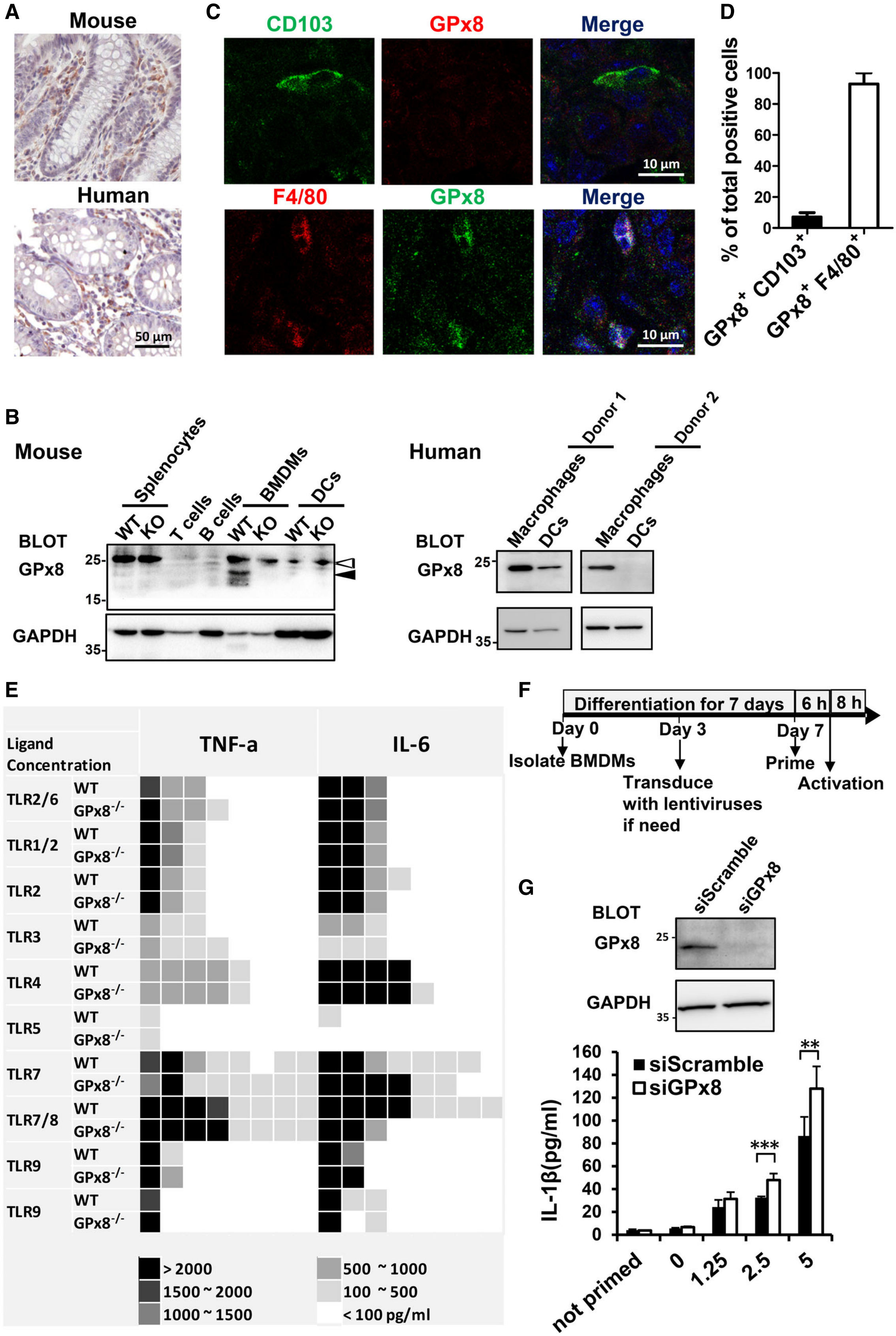

Figure EV2.

© 2019 The Authors

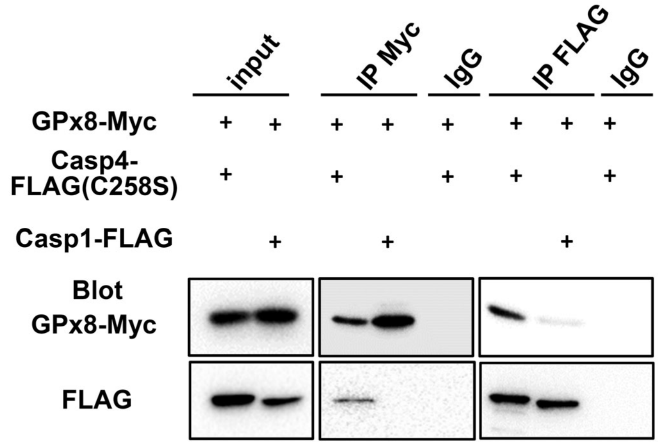

Figure EV3. GPx8 does not interact with caspase-1.

Interaction of GPx8 with human caspase-4C258S (Casp4) or human caspase-1 (Casp1) was confirmed by co-IP assays. 293T cells were co-transfected with a vector expressing FLAG-tagged Casp4C258S or Casp1, as well as a GPx8-expressing vector. 293T cell lysates containing Casp4- or Casp1-FLAG proteins were immunoprecipitated by anti-FLAG Ab and subsequently analyzed by Western blots using anti-GPx8 and anti-FLAG Ab. GPx8 and its interacting proteins were precipitated by anti-GPx8 Ab and analyzed by the indicated Abs.

Source data are available online for this figure.

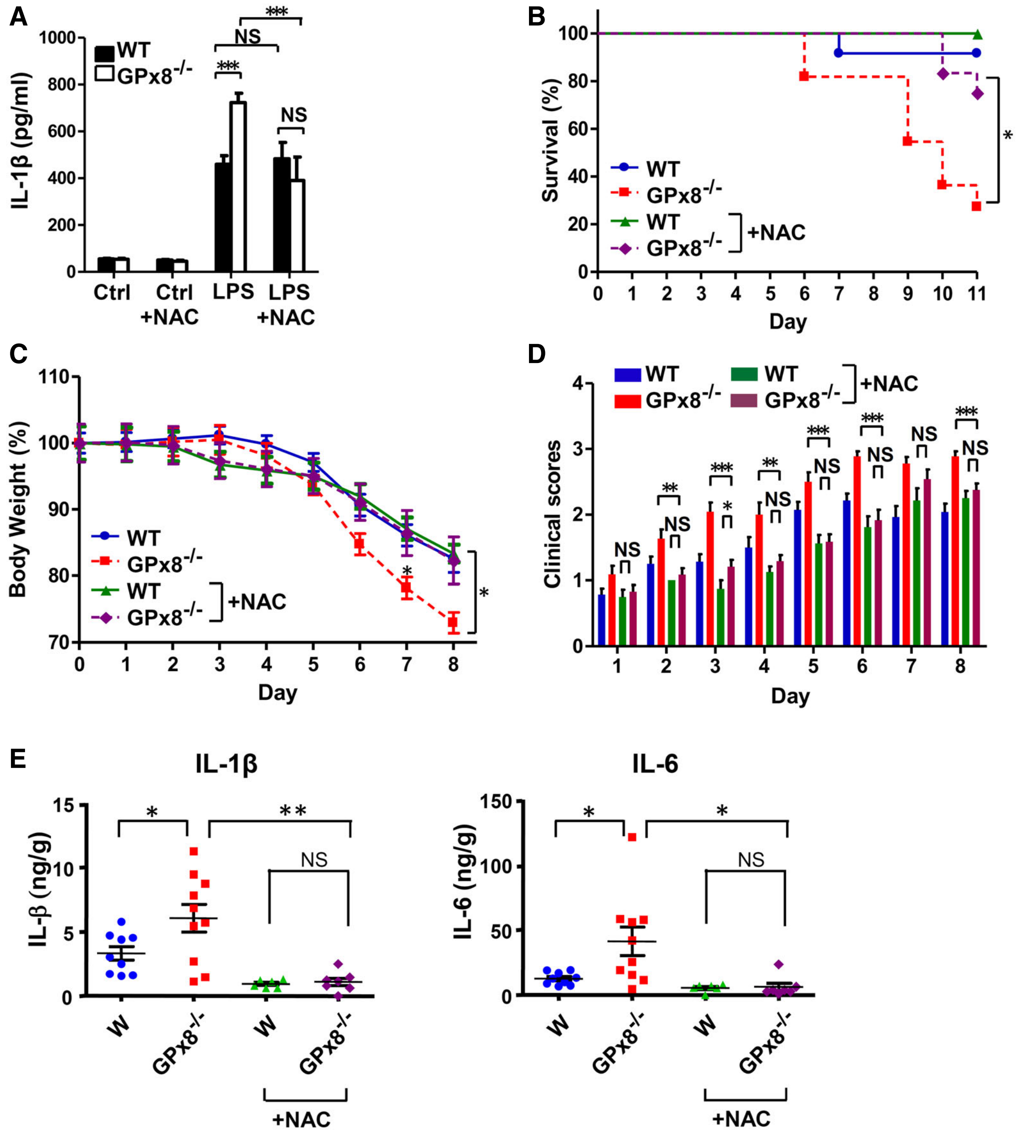

Figure EV4.

◀ **Figure EV4. N-acetylcysteine (NAC) suppresses DSS-induced colitis in *GPx8*$^{-/-}$ mice.**

A Non-canonical inflammasome activation was inhibited by NAC. BMDMs isolated from mice were primed with LPS for 5–6 h, treated with 10 mM NAC, and transfected with LPS for 12 h. Data are representative of at least three independent experiments and presented as the mean ± SD, $n = 5$, technical repeats. ***$P < 0.001$ (Student's two-tailed *t*-test).

B–E *GPx8*$^{-/-}$ mice were injected intraperitoneally with NAC (20 mg/kg). Kaplan–Meier survival plot (B), the percentage of body weight (C), and the clinical scores (D) of mice with colitis induced by 4% DSS in drinking water for 6 days ($n = 10$–11 per group). (E) Samples from mice treated with 4% DSS for 5 days were collected on day 14 ($n = 6$–10 per group). Production of IL-1β and IL-6 in colon tissue lysates. In (C–E), data were calculated using the unpaired Student's *t*-test and presented as the mean ± SEM. NS: not significant ($P > 0.05$). *$P < 0.05$; **$P < 0.01$; ***$P < 0.001$.

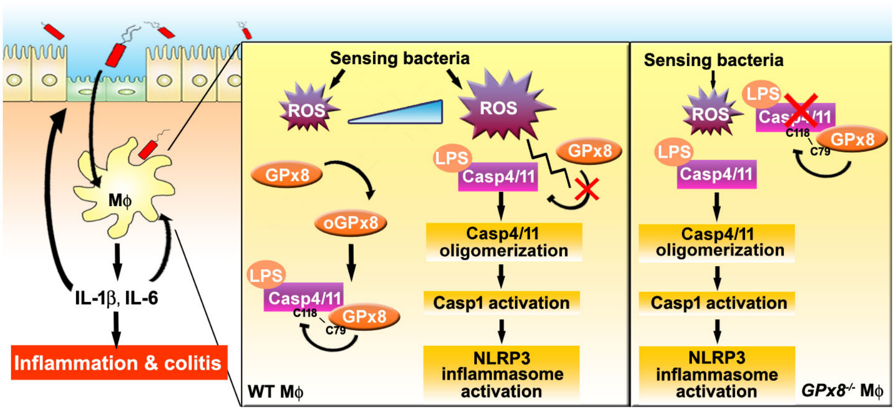

Figure EV5. A model showing how GPx8 negatively regulates caspase-4/11 and provides protection against colitis by restraining NLRP3 inflammasome activation.

When macrophages sense bacteria approaching, they become activated and produce ROS. At the beginning of activation, oxidized GPx8 covalently bonds to caspase-4 and dampens caspase-4 oligomerization to inhibit its activation. When cells begin to accumulate and produce greater levels of ROS, the GPx8-caspase-4 complex gradually dissociates and caspase-4 is activated to trigger downstream signals. In *GPx8*$^{-/-}$ macrophages, this process is exacerbated and activates the NLRP3 inflammasome, leading to the production of IL-1β and IL-6. These stimulate other macrophages or colonic epithelial cells, leading to inflammation.

© 2019 The Authors
