# Supplementary material for: Glutathione peroxidase 8 negatively regulates caspase‐4/11 to protect against colitis
Source: EMBO Mol Med. 2019 Nov 29;12(1):e9386. doi: 10.15252/emmm.201809386 (PMC6949489; doi:10.15252/emmm.201809386)

Appendix Fig S2

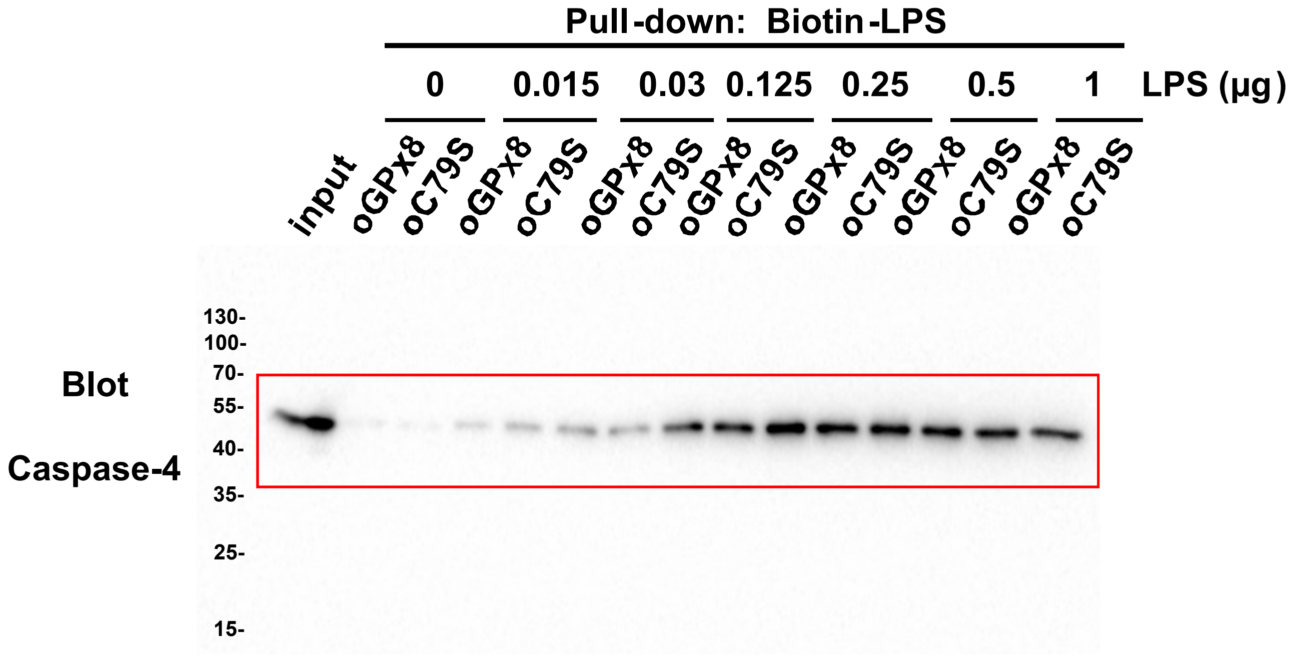

Appendix Fig S3

B

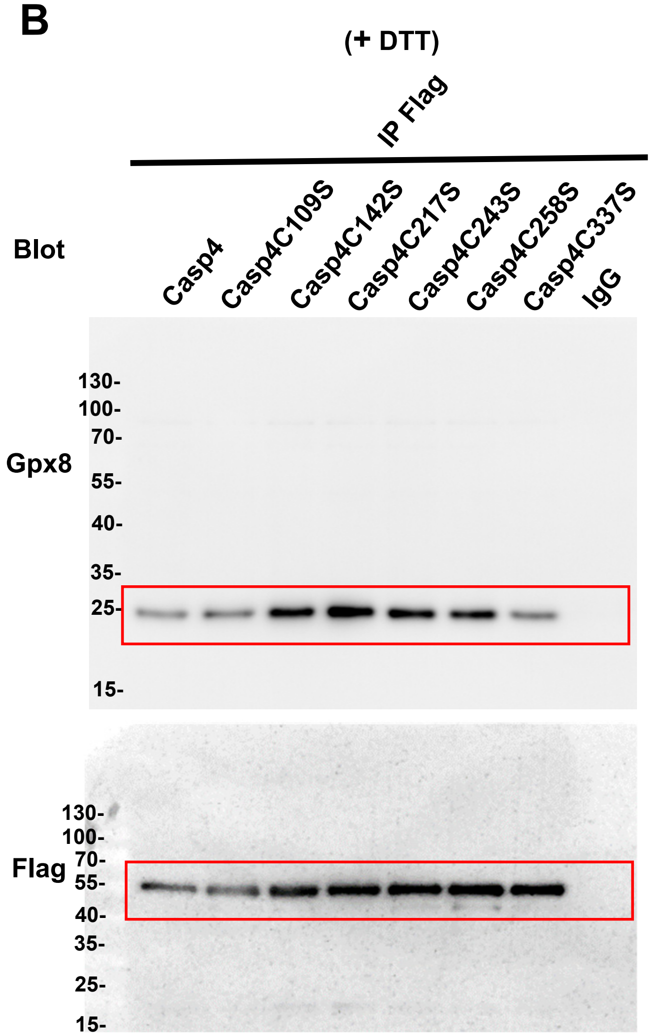

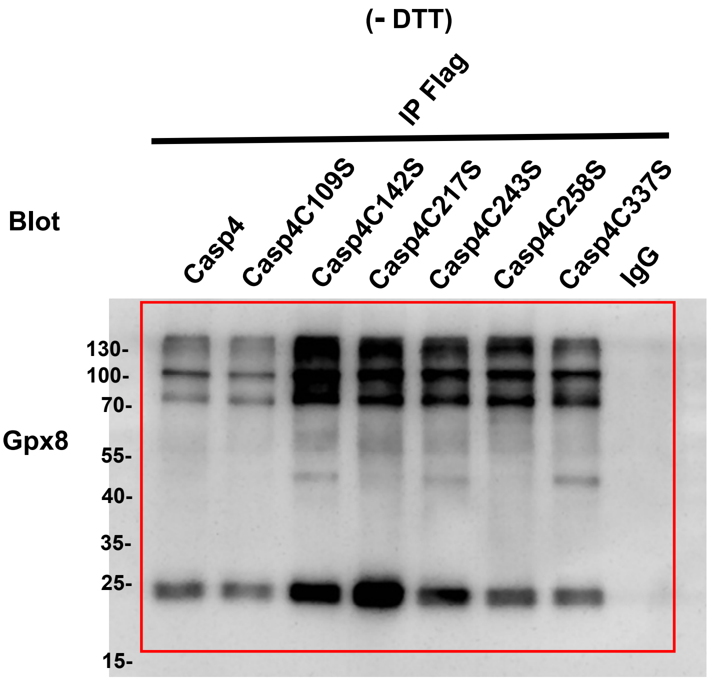

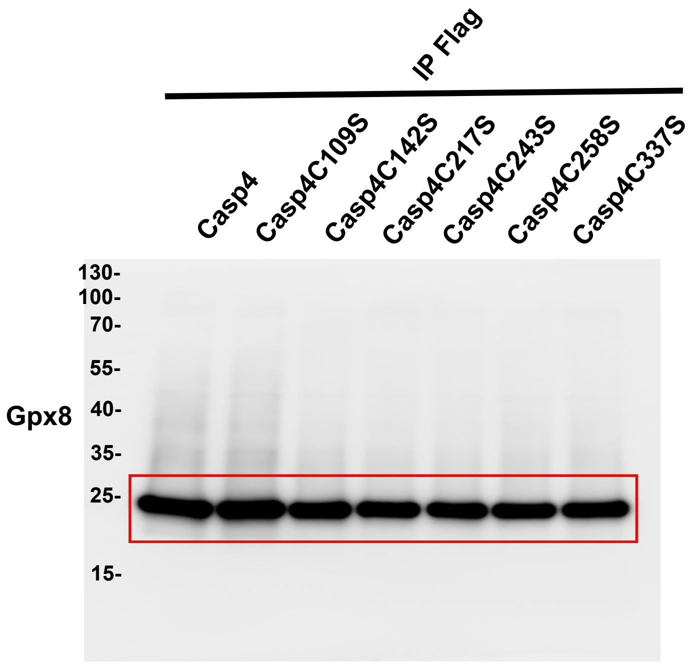

Supplement: Supplementary file 3 — Source Data for Expanded View and Appendix [file EMMM-12-e9386-s008.zip › EV_source_data/Appendix_Source_data.pdf]
