# Supplementary figures and images for: Glutathione peroxidase 8 negatively regulates caspase‐4/11 to protect against colitis
Source: EMBO Mol Med. 2019 Nov 29;12(1):e9386. doi: 10.15252/emmm.201809386 (PMC6949489; doi:10.15252/emmm.201809386)

Fig. EV1C

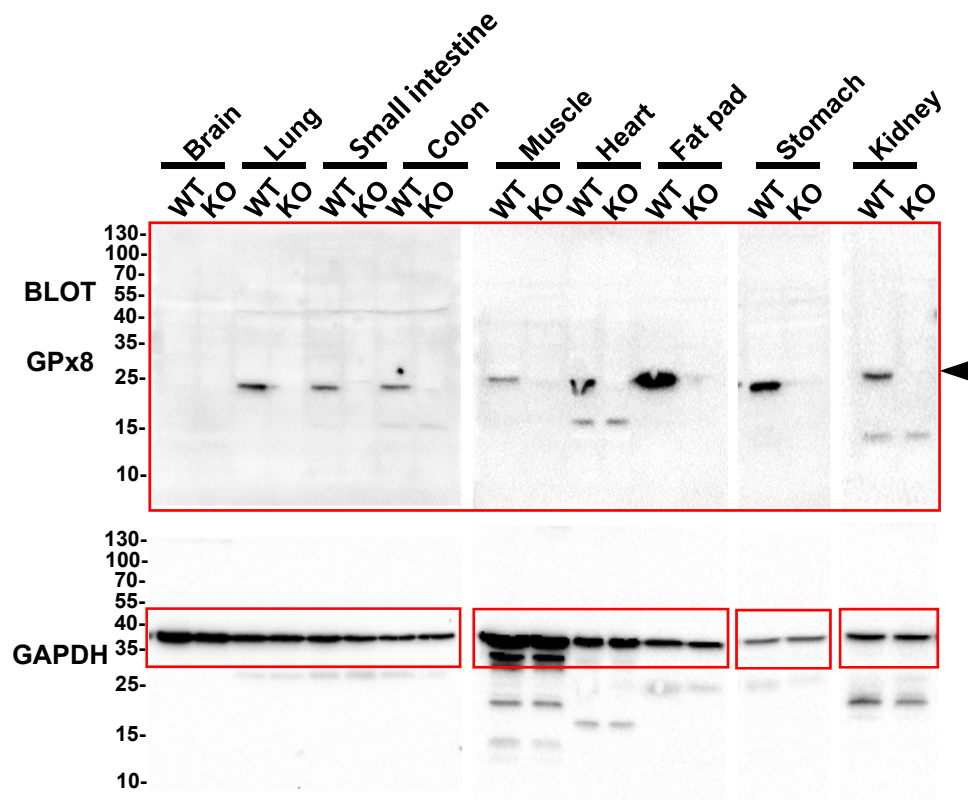

Supplement: Supplementary file 3 — Source Data for Expanded View and Appendix [file EMMM-12-e9386-s008.zip › EV_source_data/Fig_EV1.pdf]

Fig. EV2

**B**

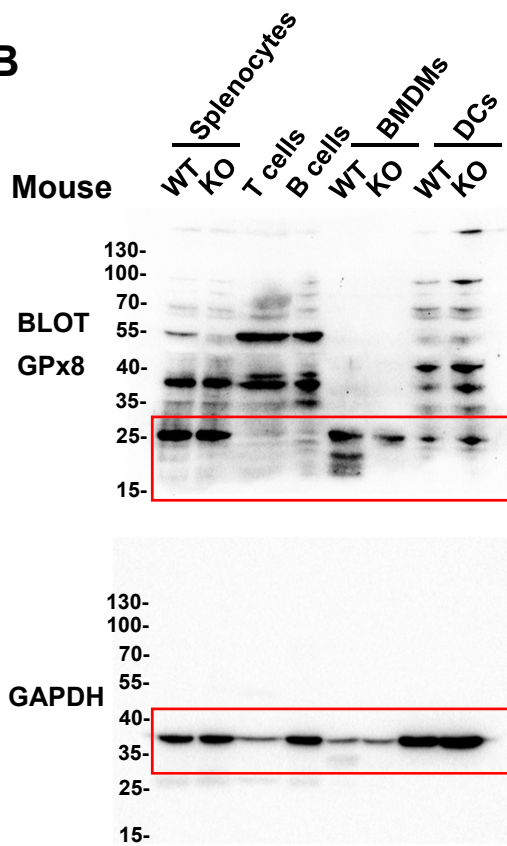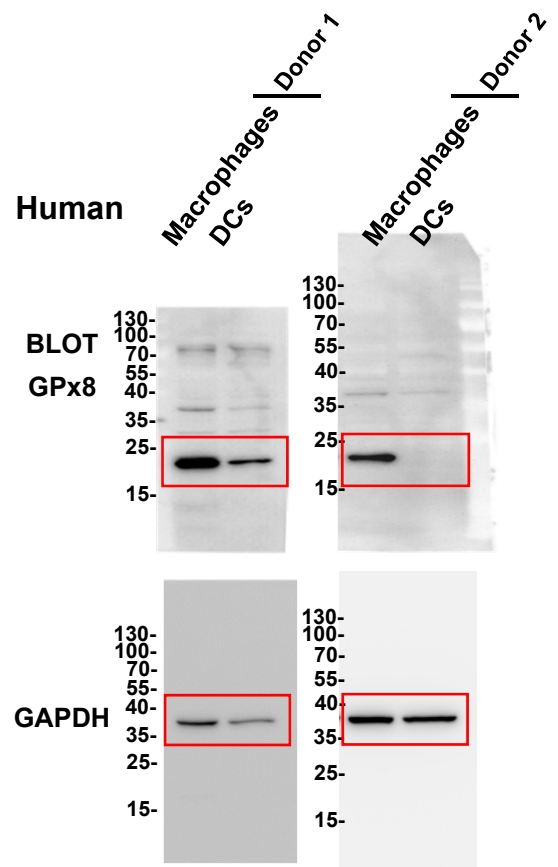

**G**

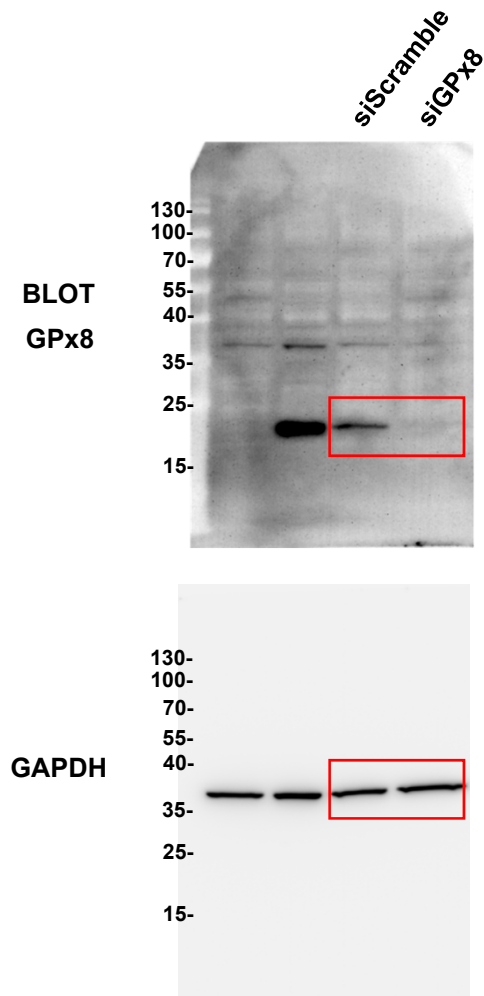

Supplement: Supplementary file 3 — Source Data for Expanded View and Appendix [file EMMM-12-e9386-s008.zip › EV_source_data/Fig_EV2.pdf]

Fig. EV3

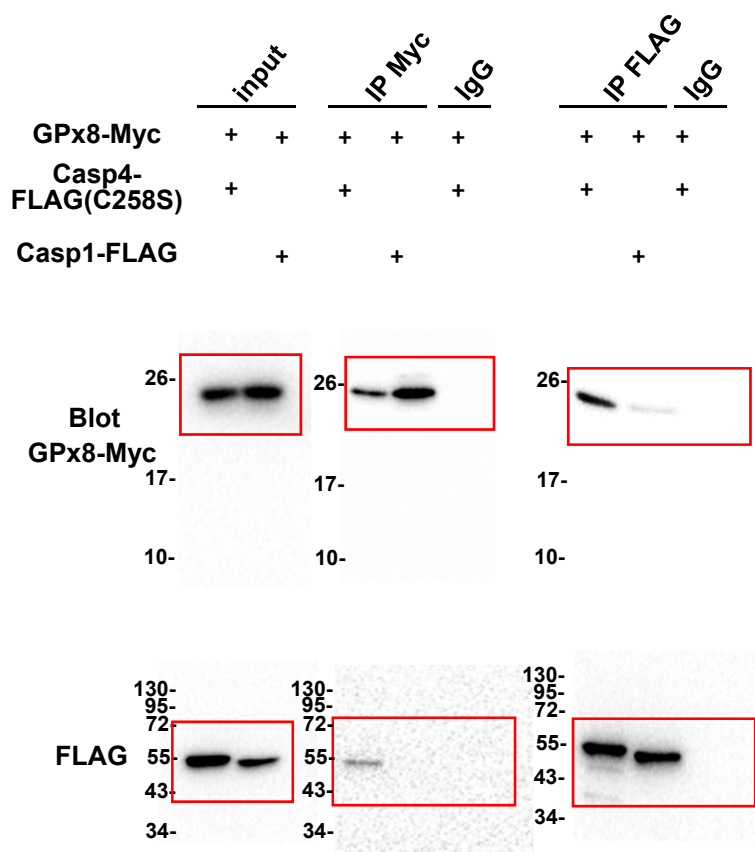

Supplement: Supplementary file 3 — Source Data for Expanded View and Appendix [file EMMM-12-e9386-s008.zip › EV_source_data/Fig_EV3.pdf]

**Fig. 3F**

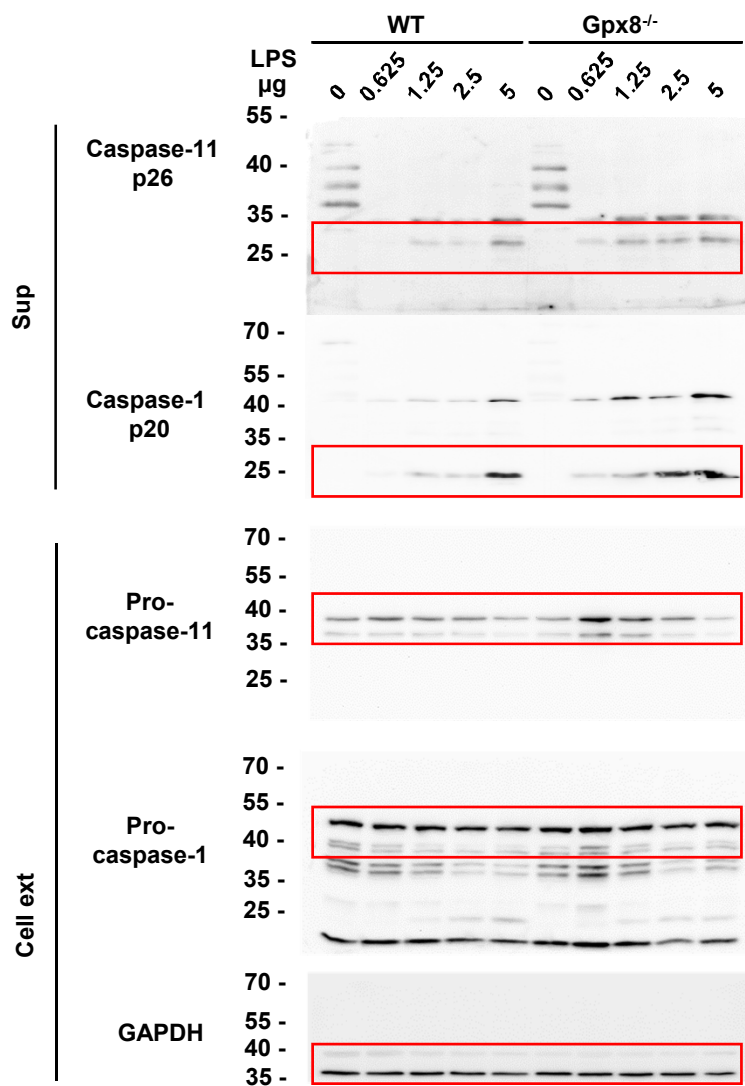

**Fig. 3H**

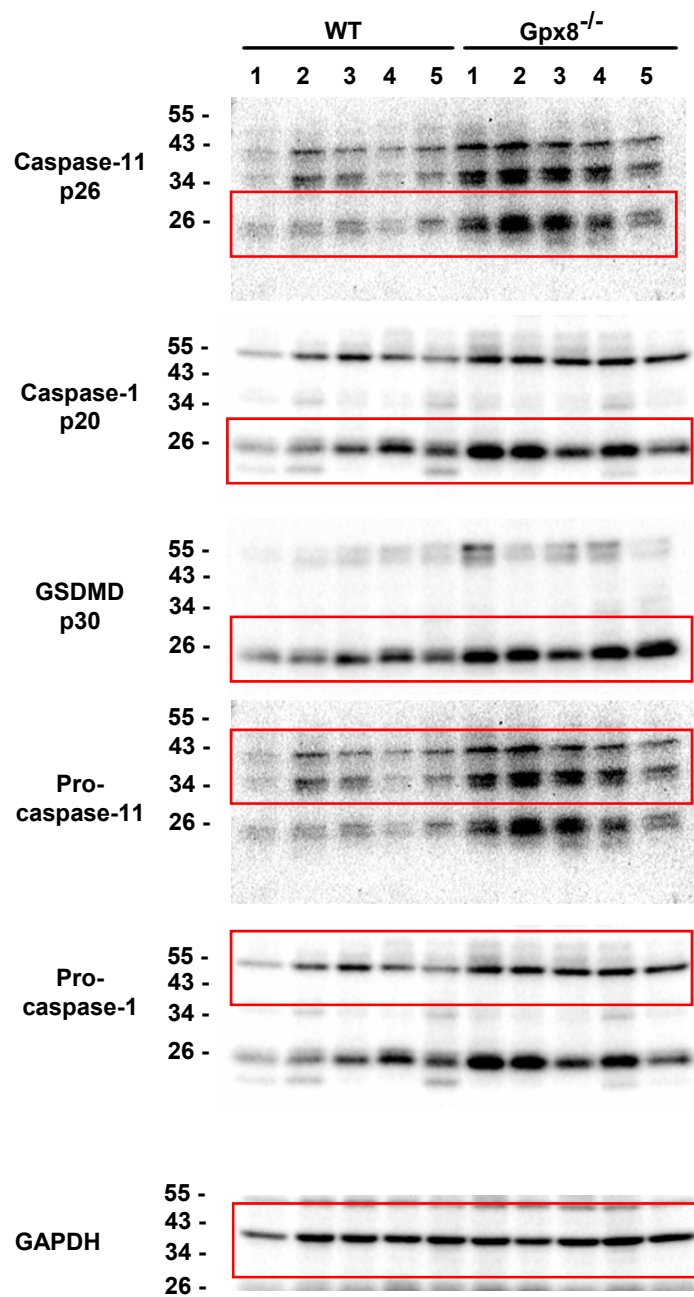

Supplement: Supplementary file 5 — Source Data for Figure 3 [file EMMM-12-e9386-s003.pdf]

**Fig. 4A**

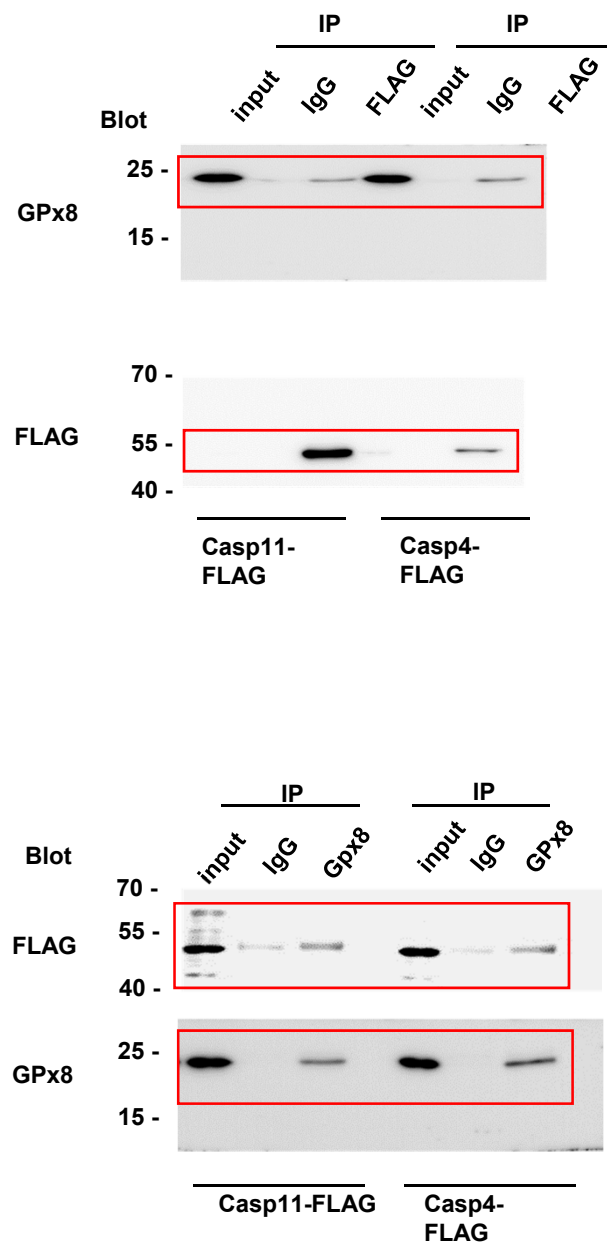

Fig. 4D

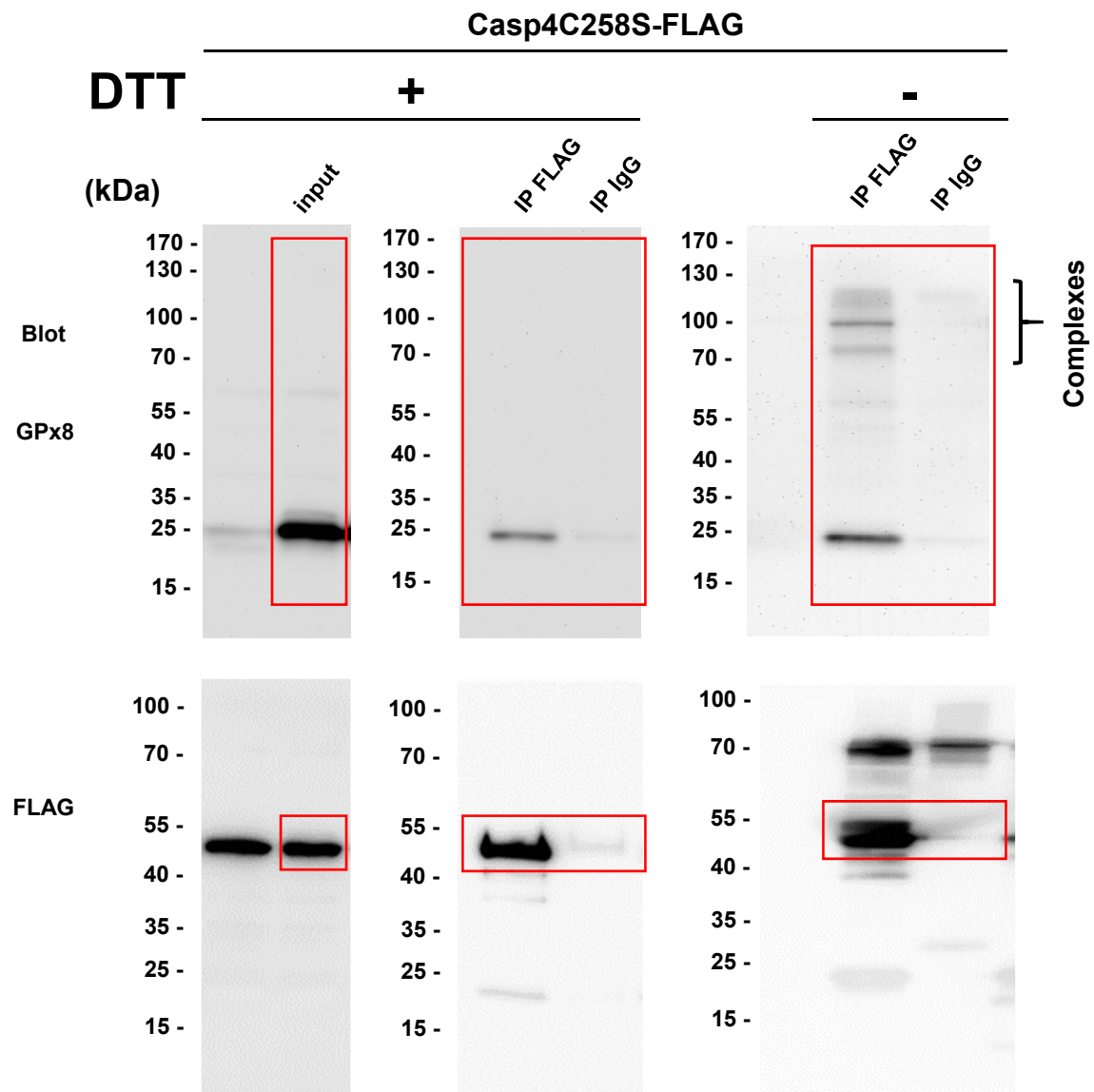

**Fig. 4E**

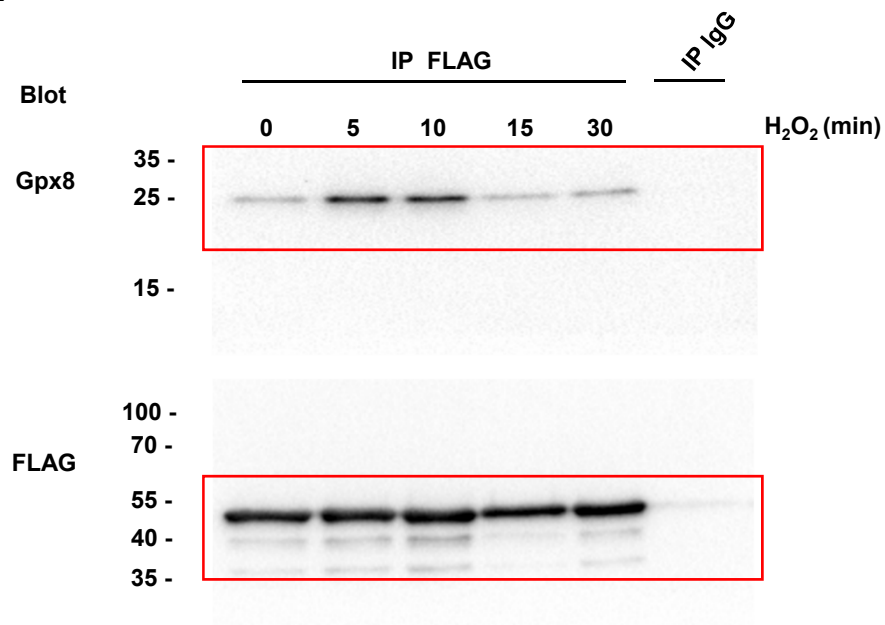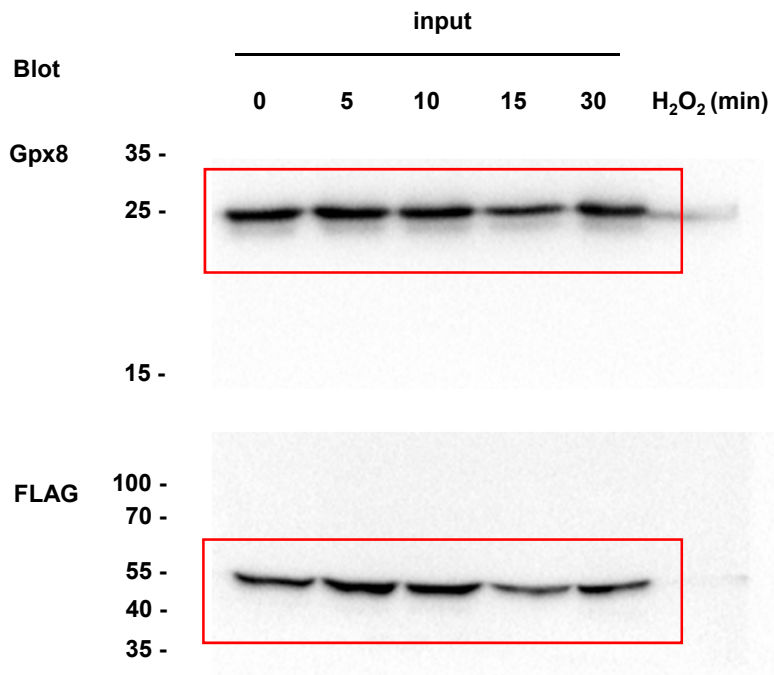

**Fig. 4F**

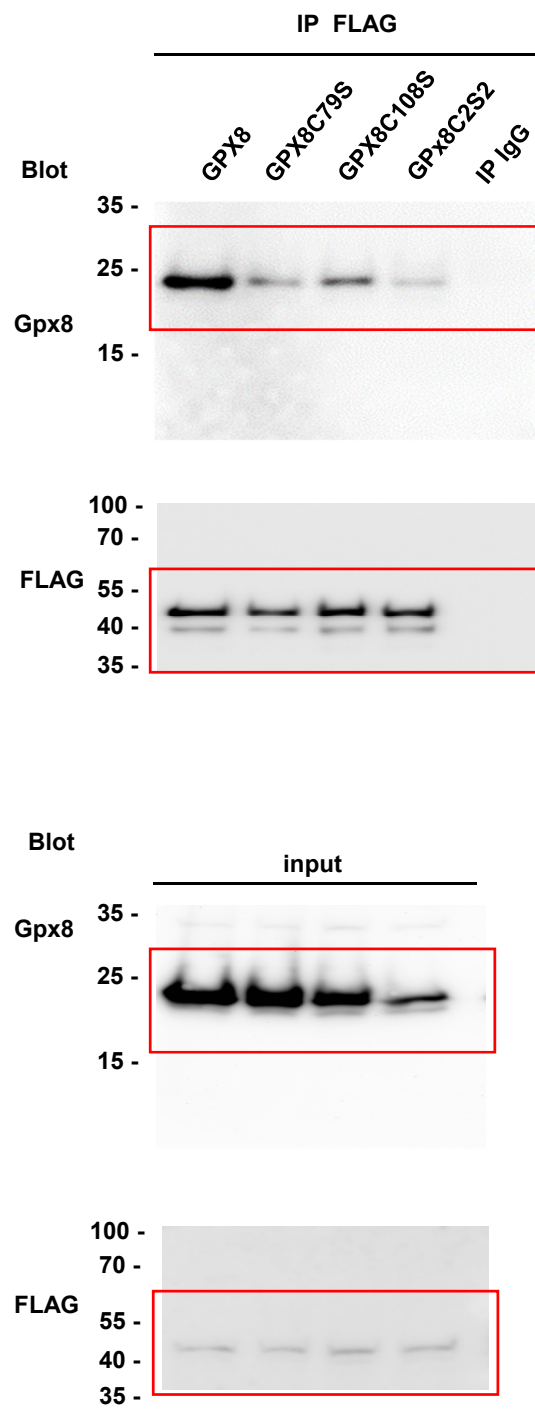

**Fig. 4G**

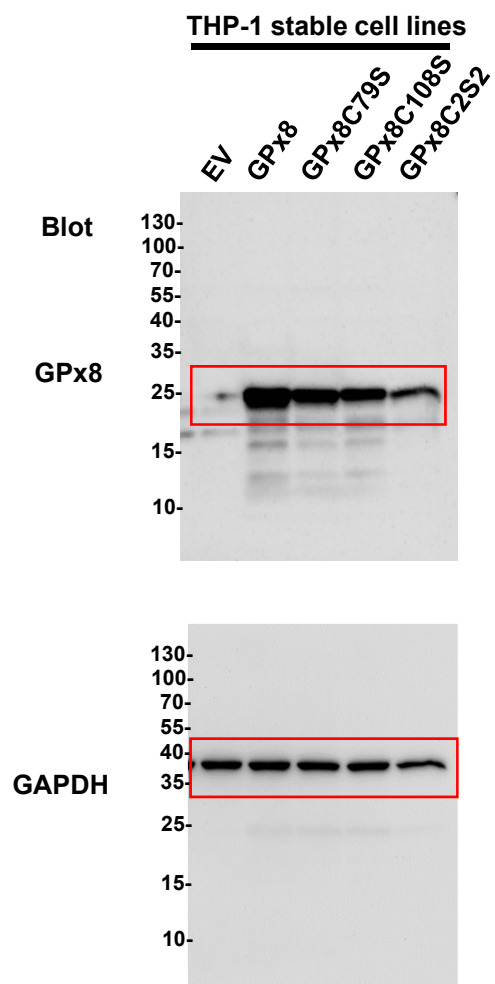

**Fig. 4J**

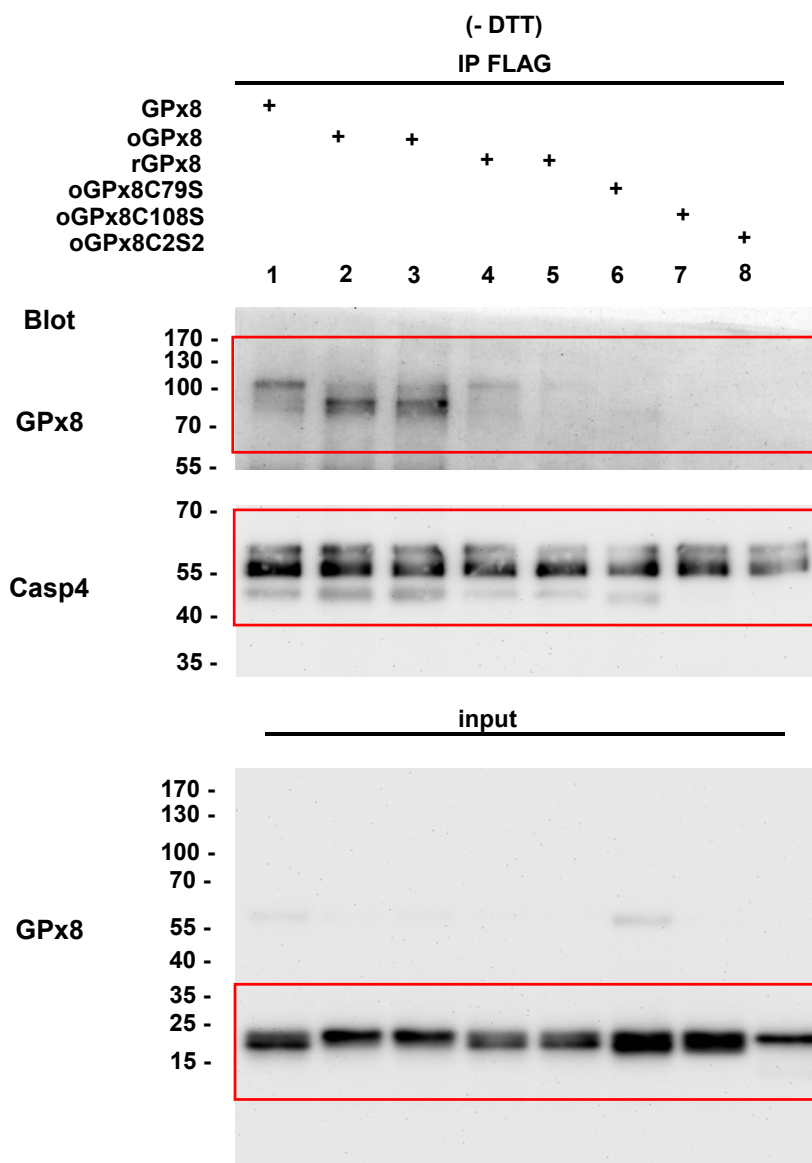

Fig. 4K

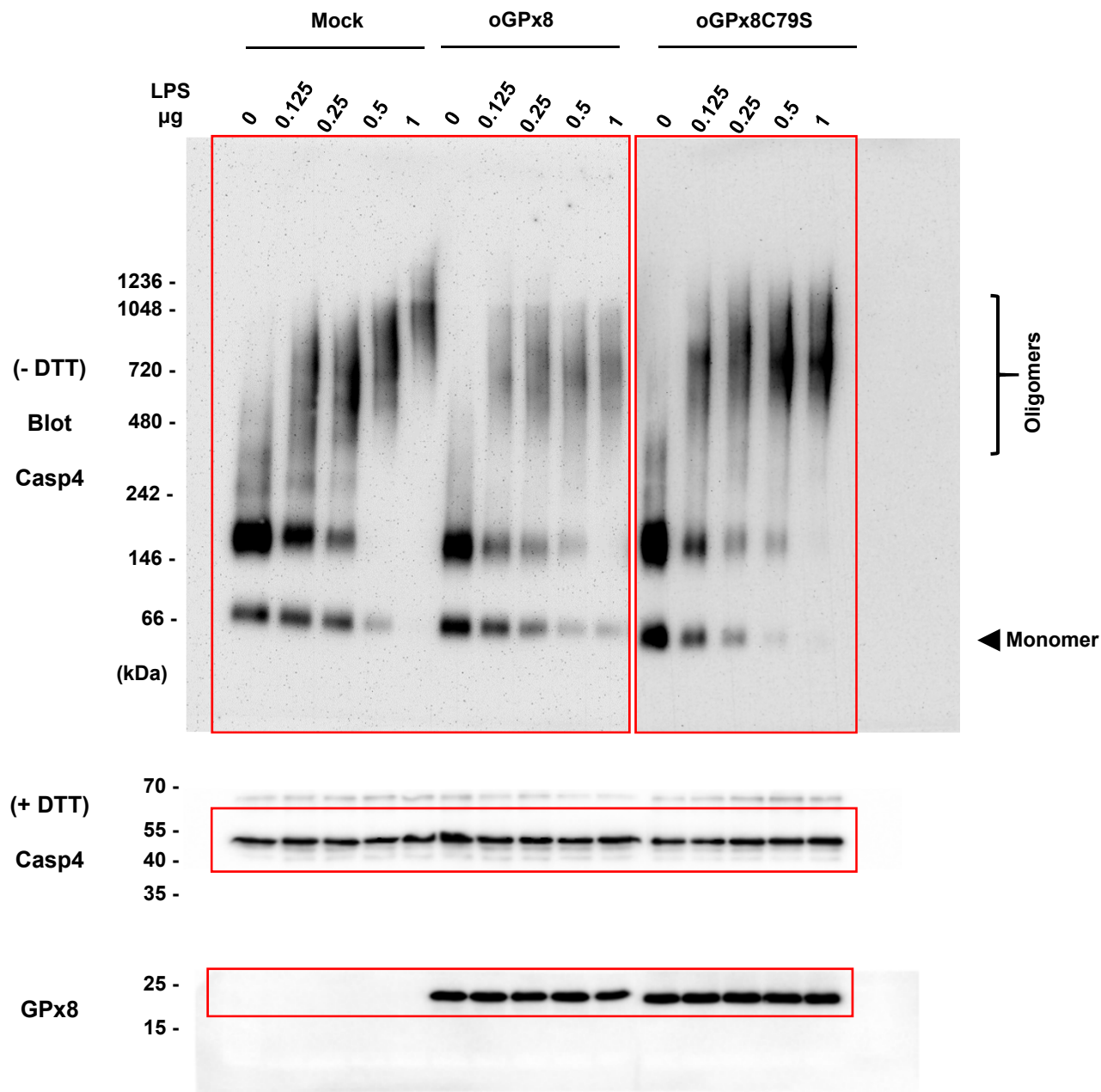

Supplement: Supplementary file 6 — Source Data for Figure 4 [file EMMM-12-e9386-s004.pdf]

**Fig. 5A**

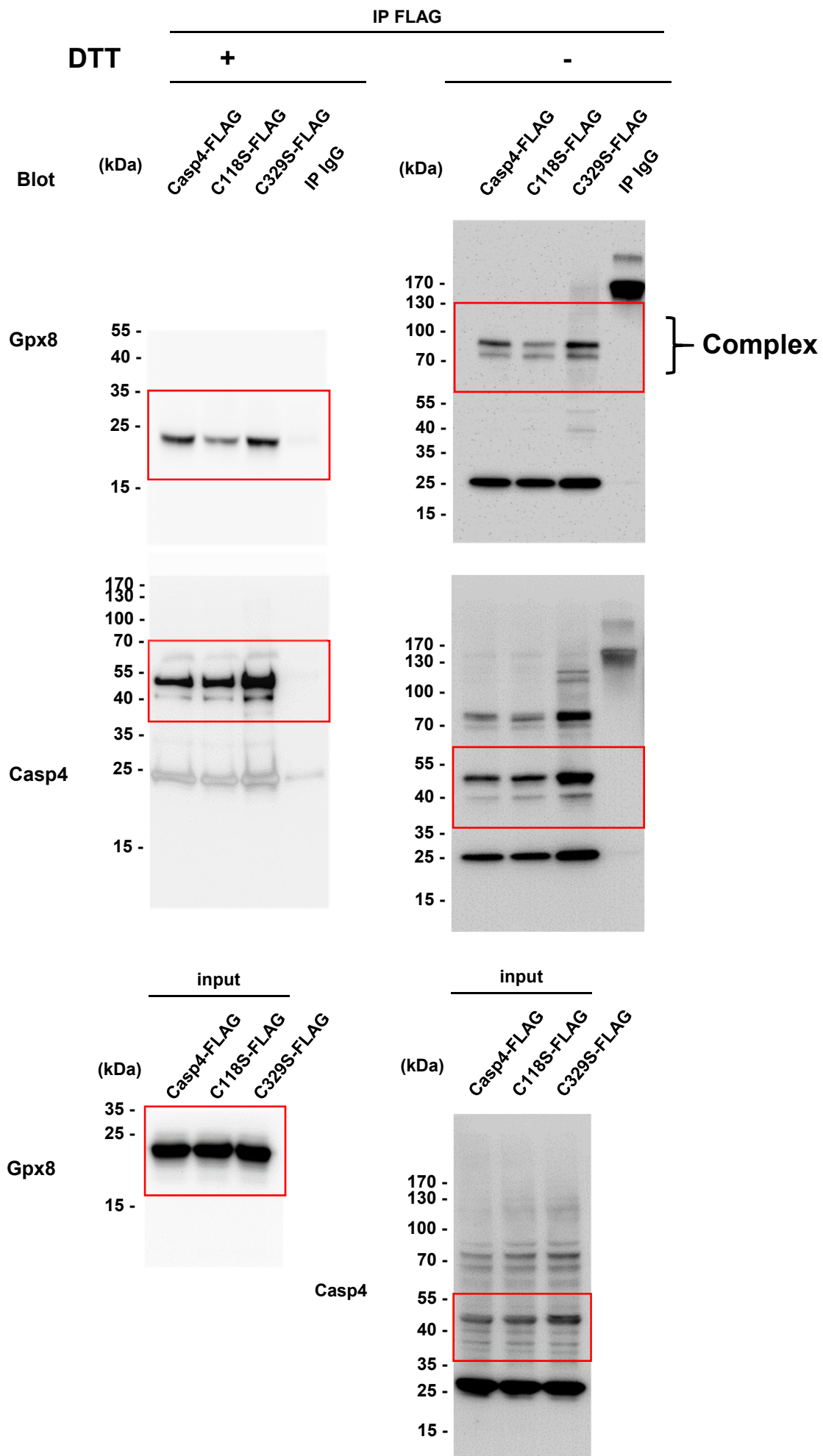

**Fig. 5B**

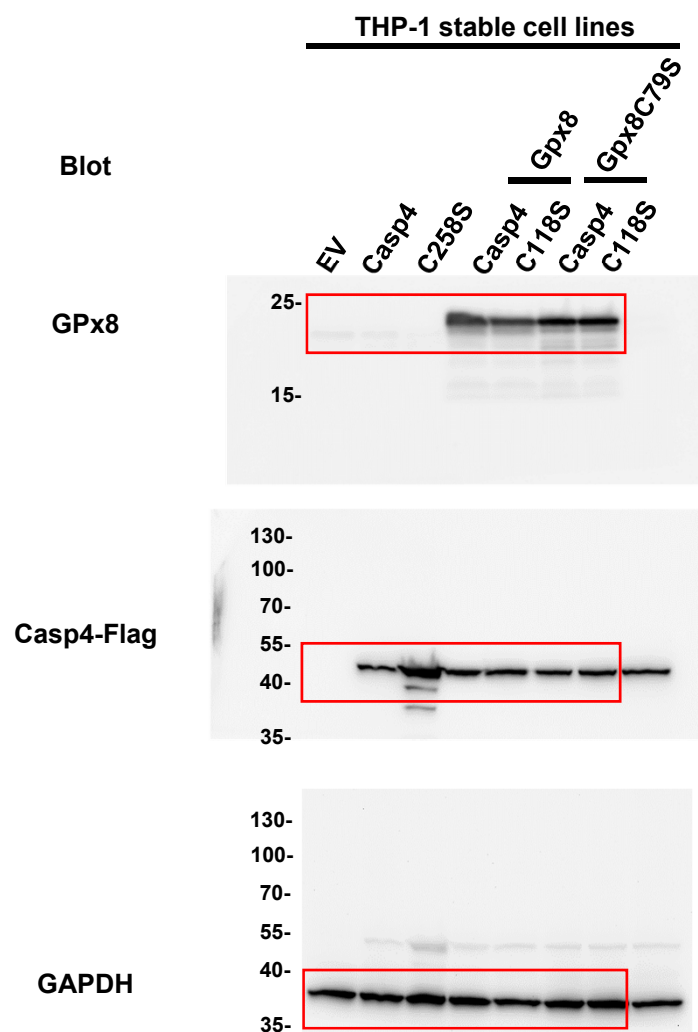

Supplement: Supplementary file 7 — Source Data for Figure 5 [file EMMM-12-e9386-s005.pdf]

**Fig. 6E**

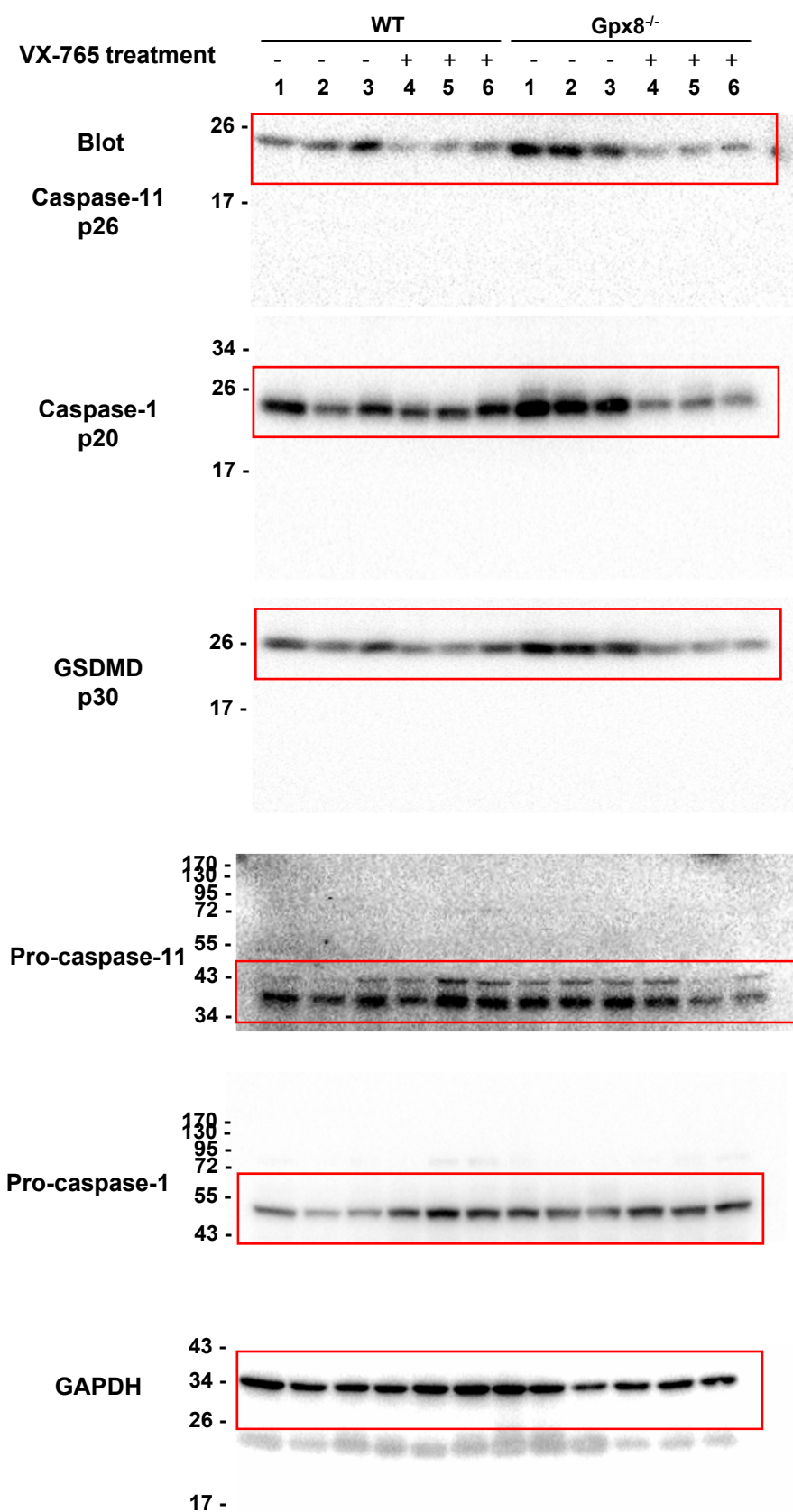

Supplement: Supplementary file 8 — Source Data for Figure 6 [file EMMM-12-e9386-s006.pdf]

**Fig. 7B**

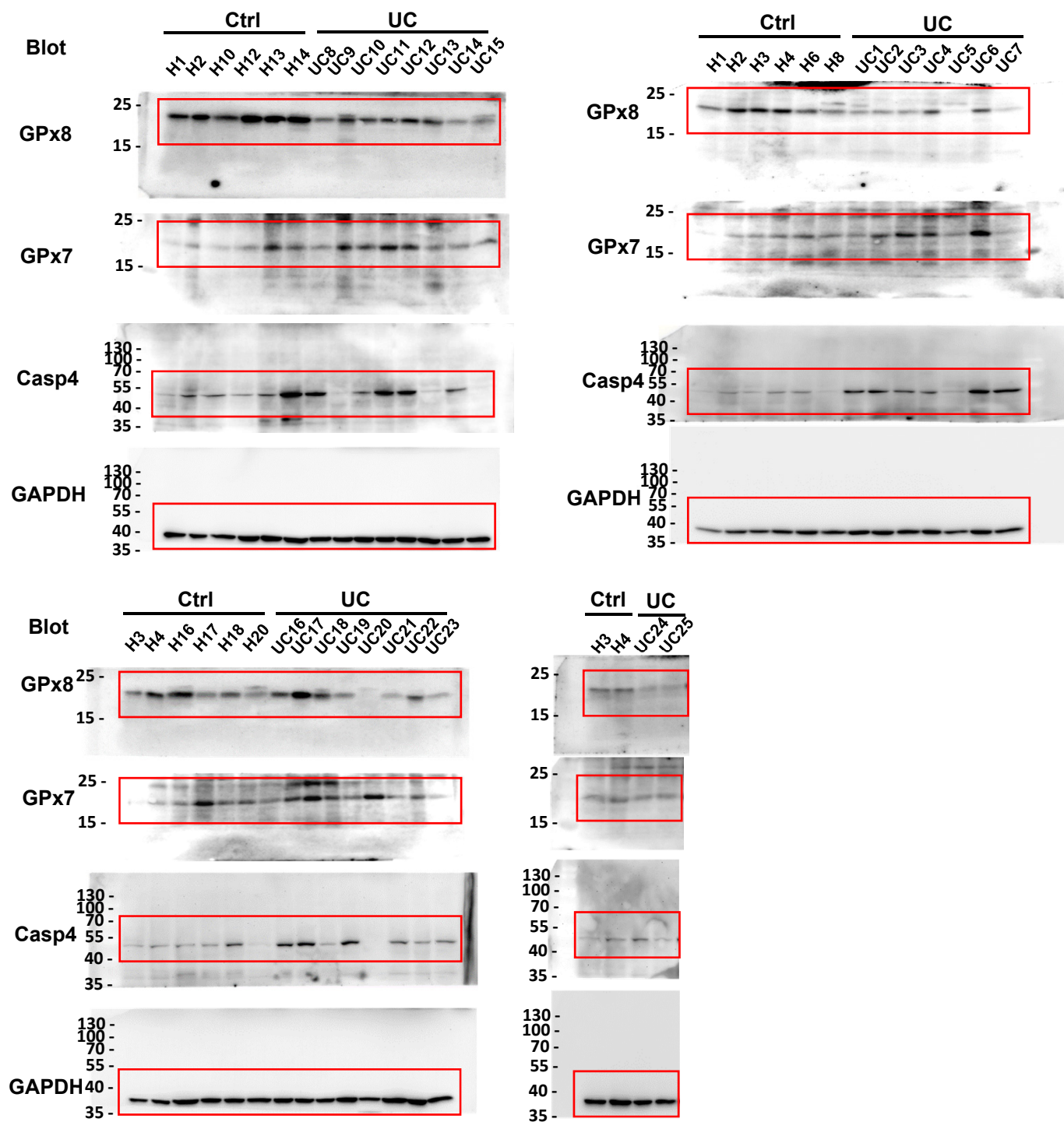

Supplement: Supplementary file 9 — Source Data for Figure 7 [file EMMM-12-e9386-s007.pdf]
